# Supplementary material for: Sigma 1 Receptor Is Overexpressed in Hepatocellular Adenoma: Involvement of ERα and HNF1α
Source: Cancers (Basel). 2020 Aug 7;12(8):2213. doi: 10.3390/cancers12082213 (PMC7464972; doi:10.3390/cancers12082213)
Supplement: Supplementary file 1 [file cancers-12-02213-s001.pdf]

Supplementary Materials

Sigma 1 Receptor is Overexpressed in Hepatocellular Adenoma: Involvement of ERα and HNF1α

Laure Villemain, Sylvie Prigent, Aurélie Abou-Lovergne, Laura Pelletier, Magali Chiral, Marco Pontoglio, Fabienne Foufelle, Stefano Caruso, Raphael Pineau, Sandra Rebouissou, Eric Chevet, Jessica Zucman-Rossi and Laurent Combettes

TTTTCTGTAGACTCTCCAGTGAGATCTCTAAGCTGGGGAGTAGGACCATTGTTTCTAGCCATATCCCTAAT
GGCCACCACAGAACTTGGCACATACAAGTGCCAACAAAGTATCTGAATGAATGACAACTCCCAGCTCTAT
CTCCAGACTGGAGCTGAGCTTCGAGACCTGGACACTCAACTGCCCACTAGACATGCAAAGGA**TGTTAGC**
CAAATAAGGGGAAAGTGGGGATAGGATAAAGGGGGAAAGAGTATTCCAGGAAGAAGAGATCAAGCCCT
GCCAACTCCATTTCCAGAGT**TGTTAAGCTCA**TTTCTTTCTTCCATCTCCACTGACACAGACCTTCCCTCTTA
CCTGGCCCACAGTCATGGTCTCCCTGACTCATCTTGCTCCCCTCCACTCGACAGTCCTGTGCCACTCGGTG
GCAAAATCTAATCCCCTTCCTCCCTTGCTGATAACCCTCAGCTGGTTTACAAGGCCTCCTCTCACATTTTGT
GCCGCAGAACAGAGAAGGAATTGTAGGCCCCTGAAGACCCCAAGCTGTCTCTCCTCGGAGGCCTTTGCTC
TAGGGGCTGGTCCCTGTGGGCTGGCATGCCCCTTCTGCGAGCTCTTAAAGACACTGCTCAGGTGTCACCGC
CTCTGGAATGCCCACCCTGGGGCTCCCAGCTTGAACTGCCTCCTCTCCGCTTCAGTCCTGGTAAAGGCGGG
CCTTAACAATCGTTGAGTCCCAGAAGGCTTCACTTCCGGGCTCGTCTGCTTCACCAGCCAGTGAGCTCGGG
CGAGCCCGTCCATTCTGGGTCTCACTATCCCCACACCTATCCGTATGTAGAGTGGCGCTAGCTAGGTCAGA
CCTCGCCTGAAGACCCTGACATCTGCCGCTGGGCGACTTGGCCCCGCCCCCTTGCCTCCCTTGCGCGGCAC
CGCCCCACTCCTCCGTGCTCCCCCCGAGGAAATGGTTCAACCGAAGGGCGGTGCCAAGGTGCCCGGGCCG
CTCCGAT**TGGTCAGGGCGAGCCGT**ACCACGGCGGTGGCGGGGGAGCGCTTC*GTGGGCAGCCGGCGGGC
TCCGAGGCCGTGAGCGCAAA***G****CCTCAGGCCCCGGCTCCCTCCTGAGCTGCGCCGTGCCAGGCCGCCCGC
CGGGATGCAGTGGGCCGTGGGCCGGCGGTGGGCGTGGGCCGCGCTGCTCCTGGCTGTCGCAGCGGTGCTG
ACCCAGGTCGTCTGGCTCTGGCTGGGTACGCAGAGCTTCGTCTTCCAGCGCGAAGAGATAGCGCAGTTGG
CGCGGCAGTACGCTGGTGAGCGGGCAGAGGGCAGGGAGGGGAGCGGCCGGCCTGGGAGCGGAGCCTAG
GGTTCCGAAGGCGCCATCCCCGGACCTAGGACCGGTGCCAGCCCTGACTCCGCCGCCCCTCTGCCTCCGC
CAGGGCTGGACCACGAGCTGGCCTTCTCTCGTCTGATCGTGGAGCTGCGGCGGCTGCACCCAGGCCACGT
GCTGCCCGACGAGGAGCTGCAGTGGGTGTTCGTGAATGCGGG

SigR1 promoter

Nucleotide sequence of the human promotor of SigR1 is shown. The major transcription start site is indicated by *. Bioinformatics analysis search tool (Mathinspector) was used to identify predicted binding sites for ERE and HNF1&.
Sequence of the putative transcription factor binding site for ERE is highlighted. Putatives transcription factor binding sites for HNF1 are in red.

Figure S1. Nucleotide sequence of the human promoter of SigR1.

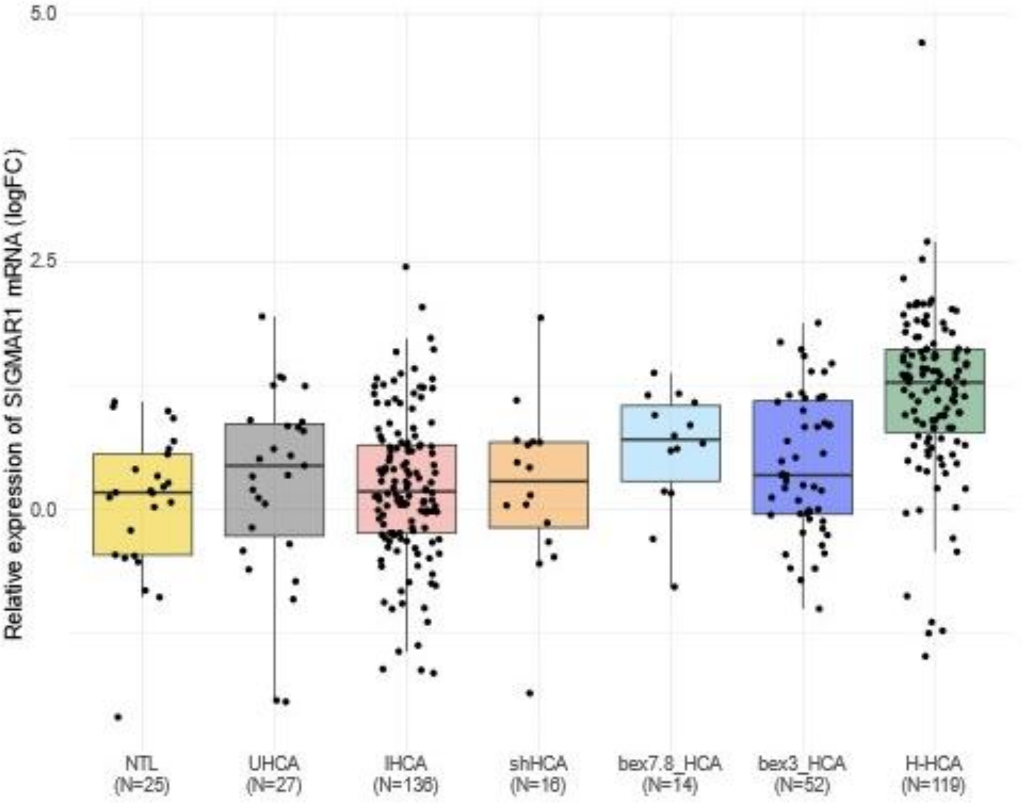

Expression of Sig1R in the different molecular subtypes of HCA:
Molecular classification of HCA was described previously in detail in (17).
Briefly, in this study we classify HCAs in 6 major subgroups:
UHCA: currently unclassified HCA according to the molecular analysis
IHCA: Inflammatory HCAs
shHCA: activation of the sonic hedgehog pathway
$b^{ex7,8}$HCA: Mutations of cadherin-associated proteinβ1 (CTNNB1) exon 7 or 8
b^{ex3}HCA: Mutations of cadherin-associated proteinβ1 (CTNNB1) exon 3
H-HCA: defined by inactivating mutations of HNF1A
Expression level of SigR1 was compared to non tumoral liver (NTL)

Figure S2. Expression of SigR1 in the different subtypes of HCA.

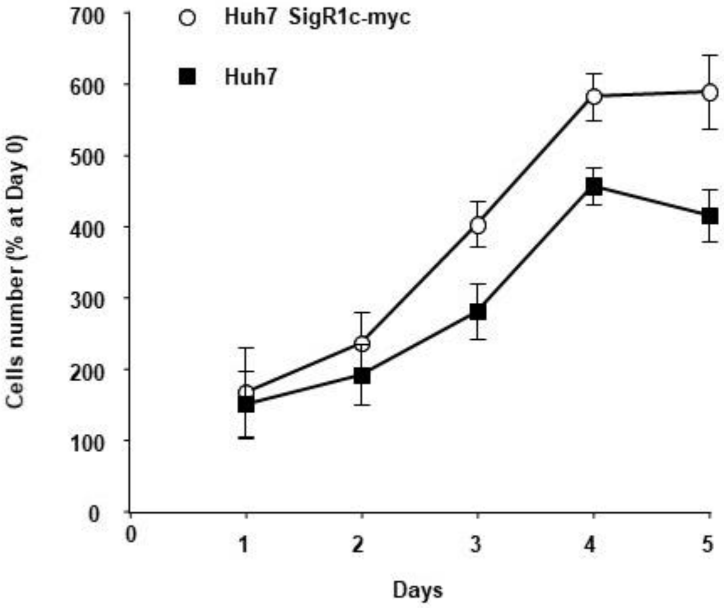

Overexpression of SigR1 increases the proliferation rate of Huh7 cells

Huh7 cells and Huh7 SigR1cMyc overexpressing SigR1 are plated in 6-well plates and counted after 1, 2, 3, 4 or 5 days. The results are expressed as the average percentage ± SEM of the number of cells at Day 0. Experiments (n=6), were performed with triplicate determinations, n > 1,000 cells per point.

Figure S3. Proliferation rate of Huh7 cells and Huh7 overexpressing SigR1.

Table S1. description of patients.

	Available data (n=391)	Number (%)
PATHOLOGY		
HCA	391	349 (89%)
HCA/HCC	391	30 (8%)
HCC on HCA	391	12 (3%)
No tumor steatosis no	347	139 (40%)
Tumor steatosis < 1/3	347	76 (22%)
Tumor steatosis 1/3-2/3	347	66 (19%)
Tumor steatosis>2/3	347	66 (19%)
MOLECULAR CLASSIFICATION		
HHCA	391	120 (31%)
$b^{ex7,8}$HCA	391	11 (3%)
$b^{ex7,8}$IHCA	391	18 (5%)
IHCA	391	137 (35%)
b^{ex3}IHCA	391	29 (7%)
b^{ex3}HCA	391	29 (7%)
shHCA	391	17 (4%)
UHCA	391	30 (8%)
GENE MUTATIONS		
HNF1A mutations*	377	122 (32%)
CTNNB1 mutations exon 3	388	52 (13%)
CTNNB1 mutations exon 7	385	21 (5%)
CTNNB1 mutations exon 8	384	8 (2%)
IL6ST mutations	386	99 (26%)
FRK mutations	380	15 (4%)
STAT3 mutations	372	9 (2%)
JAK1 mutations	374	2 (0.5%)
GNAS mutations	383	4 (1%)
TERT promoter mutations	367	8 (2%)

Table S1: patients

HCA were collected as described in (17).

*Please note that there are few samples (non-HHCA) carrying monoallelic HNF1A mutations including 7 IHCA, 1 b^{ex3}IHCA,1 b^{ex3}HCA, 1 shHCA and 1 UHCA

HCA/HCC= bordeline tumors between HCA and HCC

HCC on HCA= HCC that developed on HCA

Table S2. Primer sequences.

Gene	Sequence
SigR1 (souris)	Forward GAGAGAGGGCACCACCAAA<br>Reverse AGTAGGGTAGGTGGGACC
ERα (souris)	Forward ATGATTGGTCTCGTCTGGCG<br>Reverse GTTCCTGAAGTCTGTTGACCCT
Cyclophiline	Forward TGGAGAGCACCAAGGACAGACA<br>Reverse TAGTAACAGCTGAGGCCGT
SigR1 promoter (human)	Forward TCCGTGCTCCCCCCGAGG<br>Reverse TGGCTTTACCAACAGTACCGG

PCR primers:
Primer sequences are shown in 5′ to 3′ orientation.

Fig. 2A

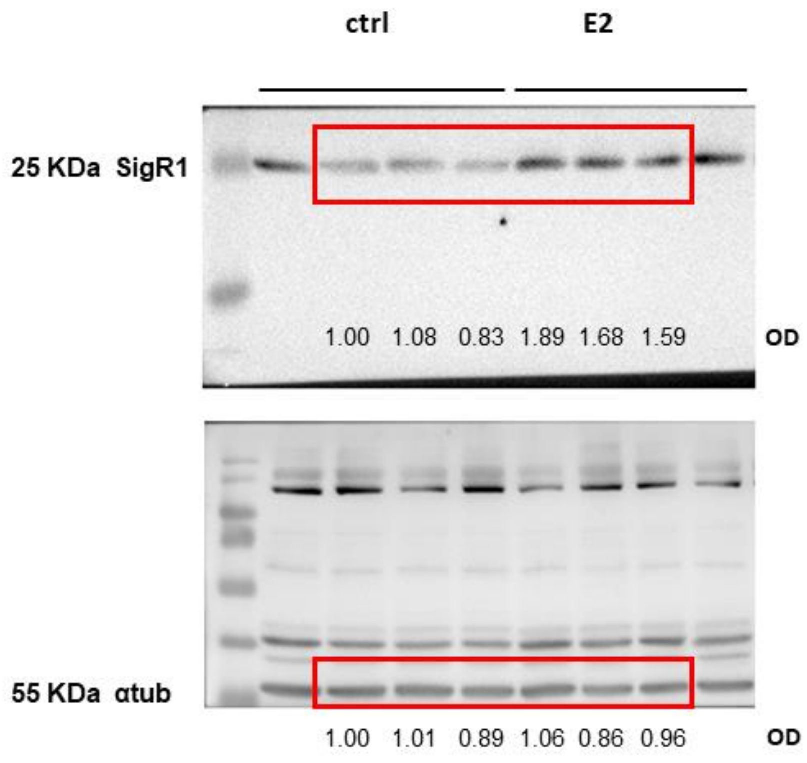

Unprocessed images of WB corresponding to figure 2A. Specific lanes presented in the final manuscript are indicated with a red box. Optical density (OD, a.u.) was determined using imageJ.

The Western blot original images for Figure 2A.

Fig. 2B

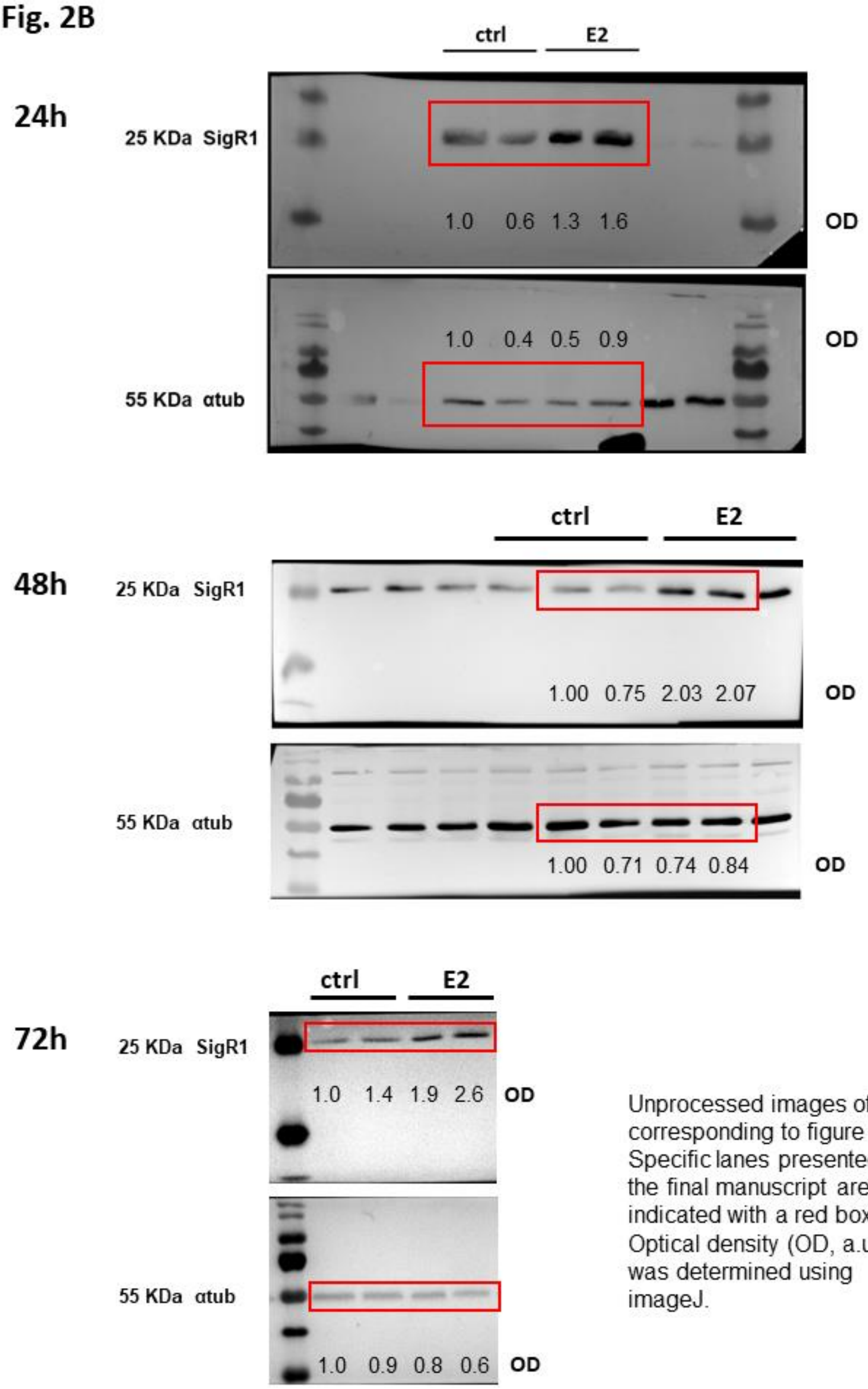

Unprocessed images of WB corresponding to figure 2B. Specific lanes presented in the final manuscript are indicated with a red box. Optical density (OD, a.u.) was determined using imageJ.

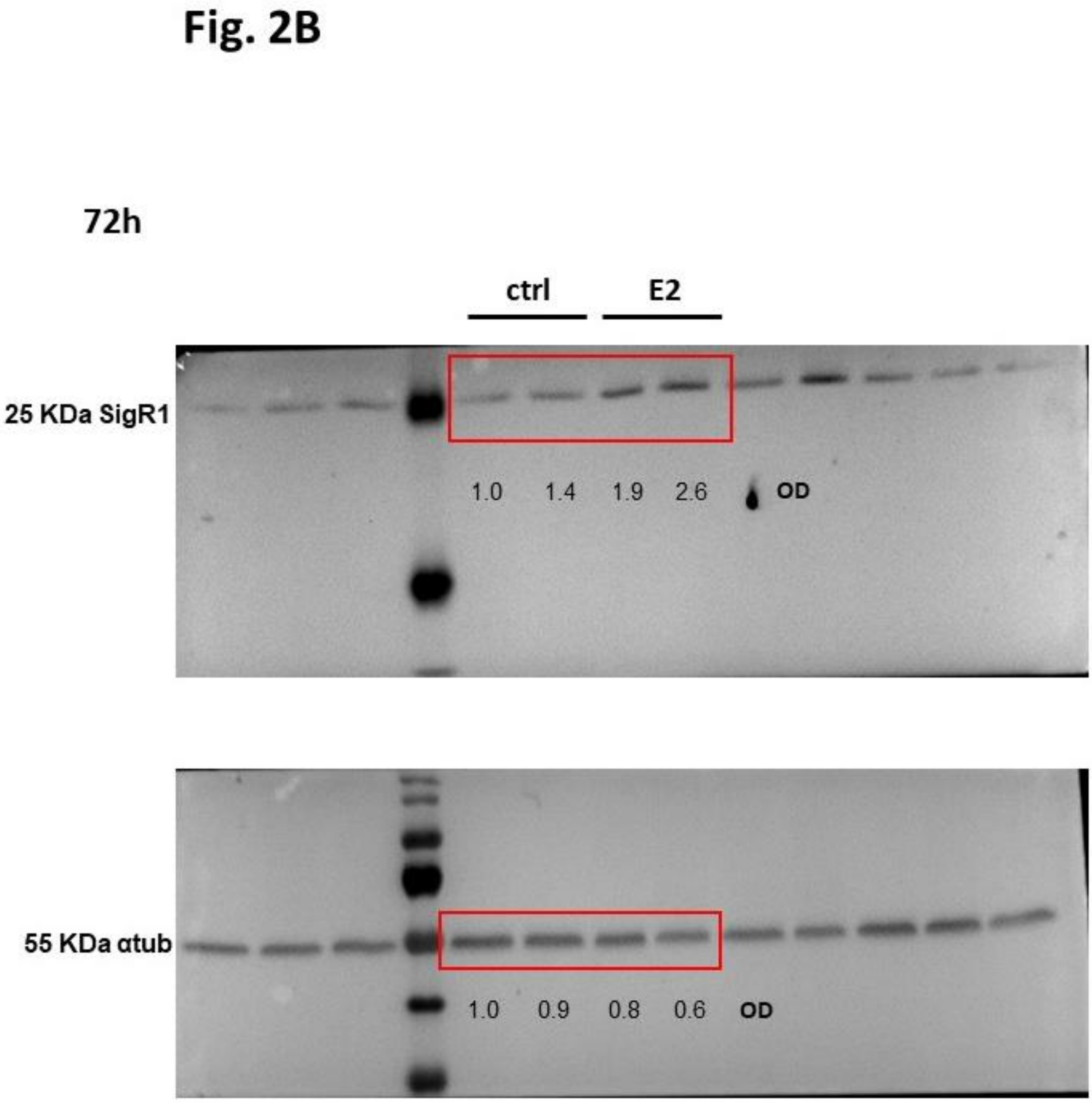

The Western blot original images for Figure 2B.

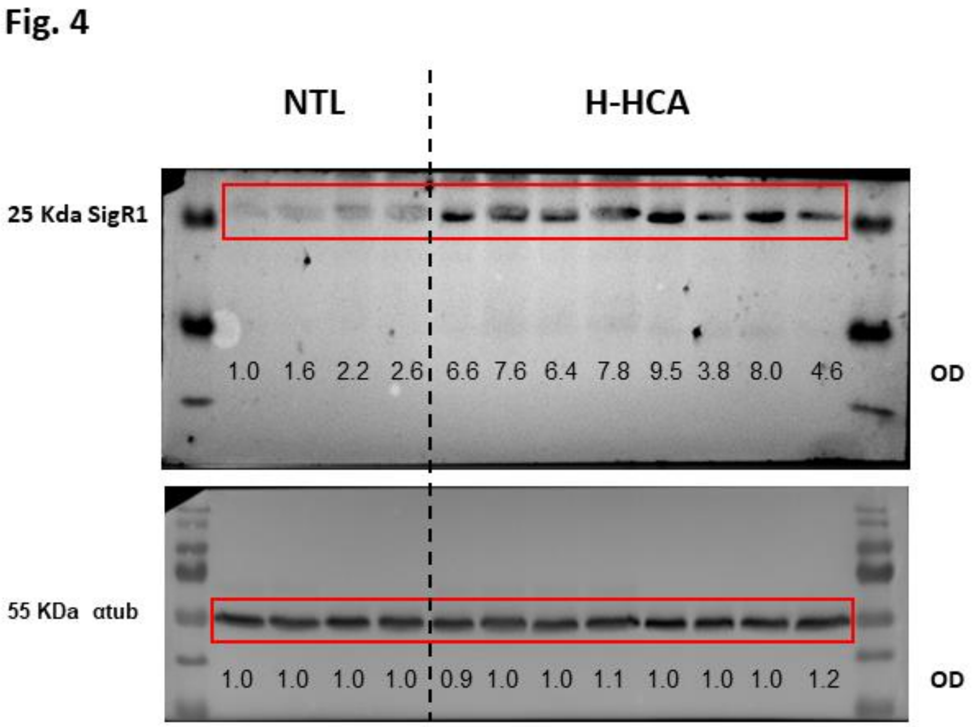

Unprocessed images of WB corresponding to figure 4. Specific lanes presented in the final manuscript are indicated with a red box. NTL: non tumor liver, H-HCA: Hepatocellular adenomas with inactivating mutations of HNF1α. Optical density (OD, a.u.) was determined using imageJ.

The Western blot original images for Figure 4.

Fig. 5B

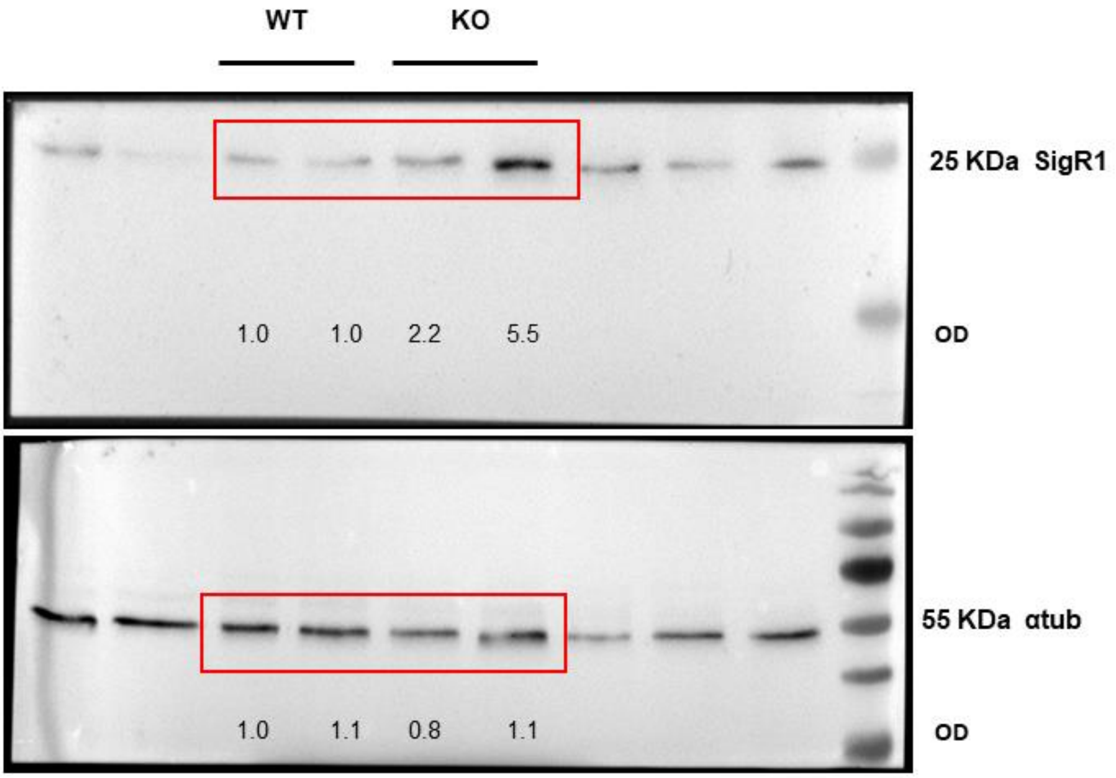

Unprocessed images of WB corresponding to figure 5B. Specific lanes presented in the final manuscript are indicated with a red box. Optical density (OD, a.u.) was determined using imageJ.

The Western blot original images for Figure 5B.

Fig. 6A

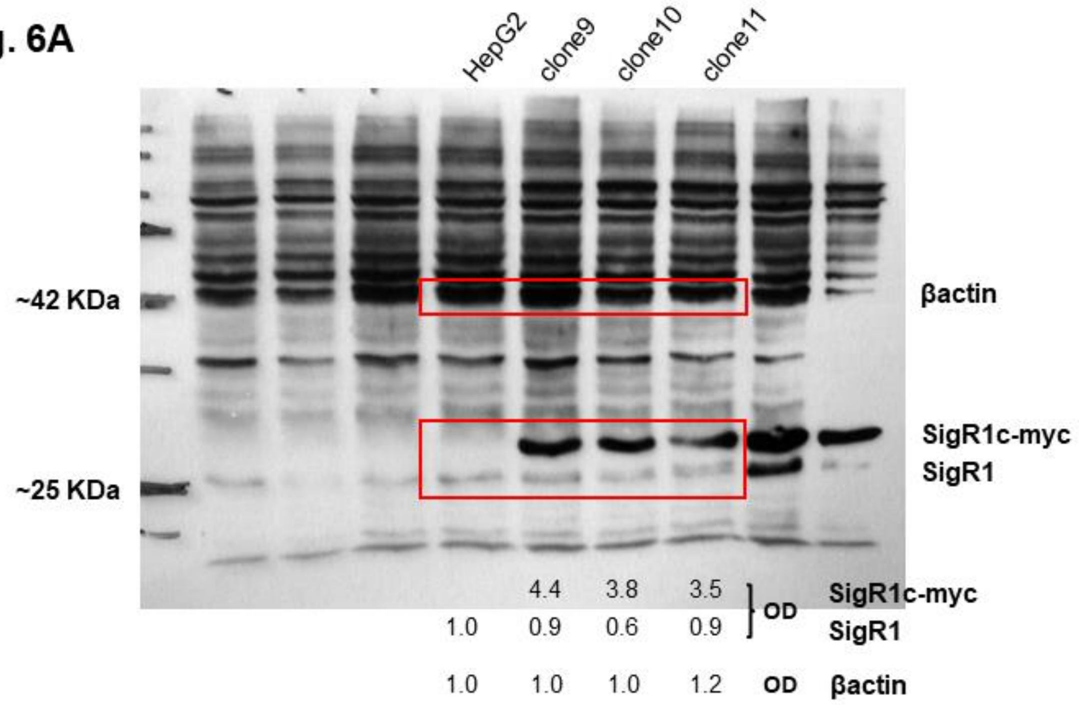

Fig. 6B

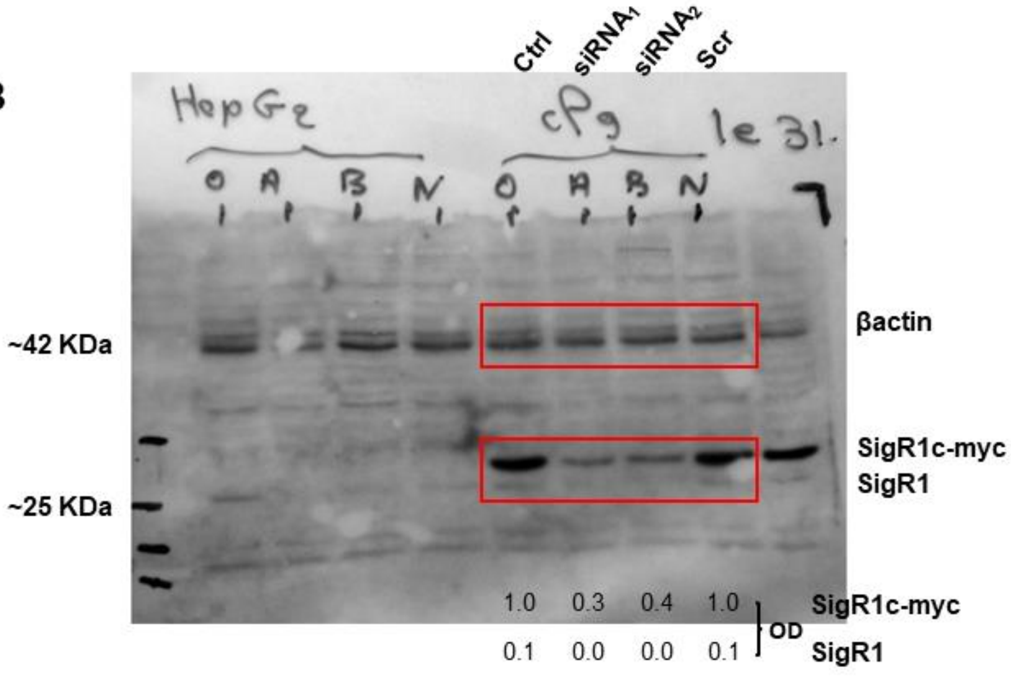

Unprocessed images of WB corresponding to figure 6A&B. Specific lanes presented in the final manuscript are indicated with a red box. Optical density (OD, a.u.) was determined using imageJ.

The Western blot original images for Figure 6A,B.

Apart from the western blots presented in figure 6 A & B which are presented under original uncropped form, all the other WBs were carried out on membranes cut following transfer. The lower part of the membrane was used for the detection of SigR1, while the upper part of the gel was used to detect alphaTub, used as loading control.

© 2020 by the authors. Licensee MDPI, Basel, Switzerland. This article is an open access article distributed under the terms and conditions of the Creative Commons Attribution (CC BY) license (http://creativecommons.org/licenses/by/4.0/).
